# Exercise-Induced Ischemic Colitis From Home-Based High-Intensity Interval Training (HIIT)

**DOI:** 10.7759/cureus.55791

**Published:** 2024-03-08

**Authors:** Benjamin J Masterman, Tanvi Ambulkar, Olivia Hartrick

**Affiliations:** 1 Gastroenterology, Southmead Hospital, Bristol, GBR; 2 General Internal Medicine, Great Western Hospitals NHS Foundation Trust, Swindon, GBR

**Keywords:** diarrhoea, lower gestrointestinal bleed, high-intensity exercise, ischemic colitis, ischaemic colitis

## Abstract

The current case presents a male in his 40s without significant past medical, surgical, or family history. Hematochezia started immediately after one hour of high-intensity interval training (HIIT), which included free-weight exercises and a circuit training instructional video. Relevant investigations included negative stool cultures and flexible sigmoidoscopy showing ischemic colitis in the mid-sigmoid. Histology also supported ischemic etiology, leading to the diagnosis of exercise-induced ischemic colitis (EIIC). The patient made a full recovery following supportive treatment, including intravenous fluid. To our knowledge, this is the first reported case of ischemic colitis secondary to HIIT performed at home. The case reviews risk factors for EIIC and highlights the diagnosis as being possible outside the context of long-distance and endurance exercise.

## Introduction

Ischemic colitis is a term used to encompass the inflammatory process in the gut that results from insufficient blood supply and subsequent deprivation of oxygen. It is commonly associated with vascular risk factors such as hypertension, smoking, age, and peripheral vascular disease, or thromboembolic diseases such as cardiac arrhythmias and coagulopathy [1]. Severe cases of ischemic colitis carry significant mortality and may present with septic shock, requiring surgical intervention [2,3].

Transient or reversible ischemic colitis is often less severe, presenting with nonspecific symptoms such as abdominal pain, diarrhea, and hematochezia. Symptoms may rapidly resolve, and diagnosis depends on characteristic appearances at endoscopy and histopathology [1].

Exercise-induced ischemic colitis (EIIC) is a form of transient ischemic colitis linked to endurance exercises like marathon running and long-distance cycling [4-8]. Pathophysiology is thought to be related to the sympathetic nervous system activating a catecholamine surge. This results in splanchnic circulation vasoconstriction and hypoperfusion of the gut, making it prone to ischemia [7,9]. Increased blood viscosity, and therefore dehydration, also plays a role in increasing susceptibility [10]. Traditionally, EIIC occurs in “watershed” areas of the colon, such as the splenic flexure and sigmoid, thought to be the most at risk of ischemia due to their end arterial supply [3,9].

Increasingly, following the COVID-19 pandemic, exercise performed at home has become an important alternative to fitness activities performed in shared spaces such as gyms [11]. High-intensity interval training (HIIT), a form of exercise that involves short bursts of high-intensity activity (e.g., sprinting) interspersed by periods of rest, is associated with numerous health benefits, including improvement in stamina [12] and reduced risk of cerebrovascular events [13]. Although there are no case studies linking HIIT or circuit training with EIIC, the pathophysiological mechanisms of prolonged intense exercise leading to gut hypoperfusion are likely transferable between different forms of endurance exercise, especially if rest periods are short.

## Case presentation

A male in his forties presented to the emergency department with acute-onset hematochezia and diarrhea, which started during and immediately after a period of HIIT at home. There was no significant past medical, surgical, family, or smoking history. The patient did not take any regular prescribed or over-the-counter medications. Before exercising, the patient ate a breakfast of Weetabix (R), semi-skimmed milk, and low-fat yogurt at 8:30 am, then two Nespresso pods (~120 mg caffeine) between 8:45 and 9:30 am. There was no fluid hydration other than this throughout the morning. The HIIT started at 9:00 am and included intensive resistance training, including free weights ranging from 5 to 35 kg, and a circuit training instructional video hosted online. The highest recorded heart rate was 190 bpm with a mean range of 165-170 bpm without cardiac symptoms, measured using the Polar H9 (R) with the corresponding Android app. The patient regularly participated in similar exercise sessions; however, relevant differences on this occasion were exercising earlier in the morning, for a longer duration, and without breaks.

Loose stools developed toward the end of exercise and continued post-exercise. This transitioned into frank per rectum bleeding and lethargy in the absence of fever.

Investigations

**Figure 1:**
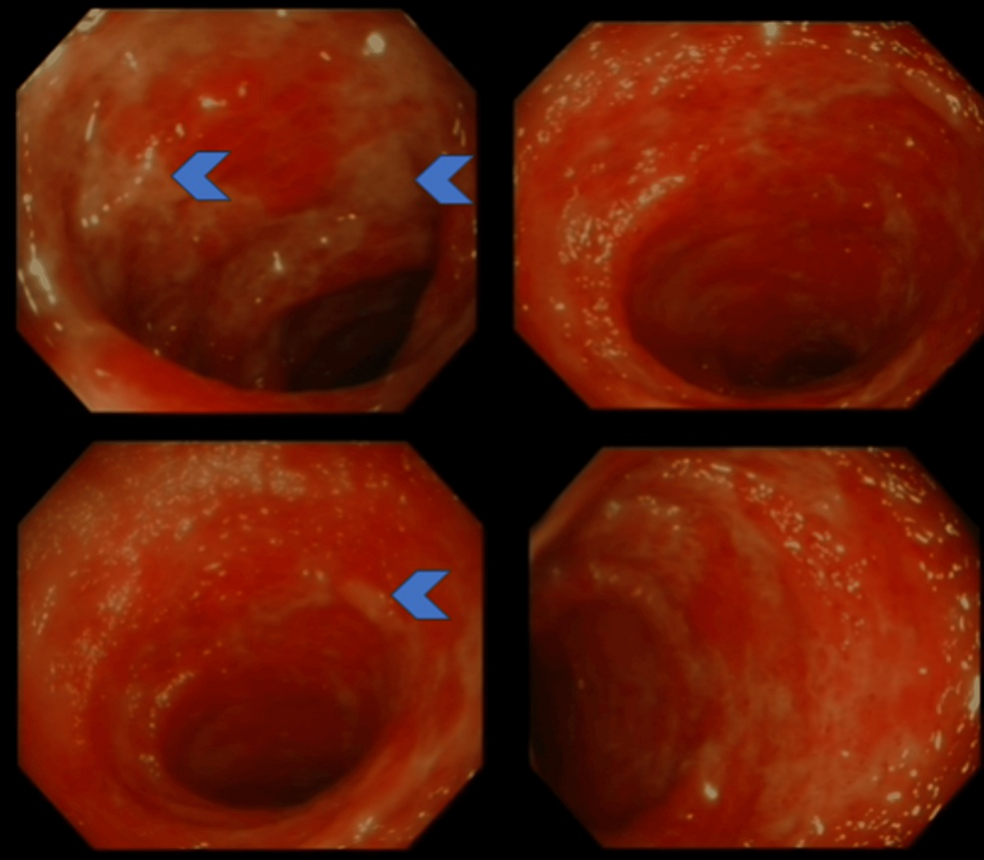
Images obtained during flexible sigmoidoscopy Confluent colitis is evident from generalized erythema and loss of vascular pattern. This is interspersed by an overlying fibrinous exudate (arrowheads), which starts in the mid-sigmoid.

The admission ECG demonstrated sinus tachycardia with no evidence of atrial fibrillation. Blood tests were performed, which revealed leukocytosis with a white cell count of 17.5 × 109/l (4.0-11.0) and an elevated neutrophil count of 14.4 × 109/l (2.0-7.5). The C-reactive protein was mildly elevated at 8 mg/L (<5) with preserved renal function. Liver function test results returned within normal limits. Stool MC&S and *Clostridium difficile* were normal and did not reveal any growth. An X-ray of the abdomen revealed potential thickening of the mucosa and gas in the left upper quadrant. A CT angiogram was not requested due to a suspected initial diagnosis of infection and subsequent clinical improvement. Given ongoing bleeding for 24 hours, a flexible sigmoidoscopy was performed as an inpatient (Figure 1).

**Figure 2:**
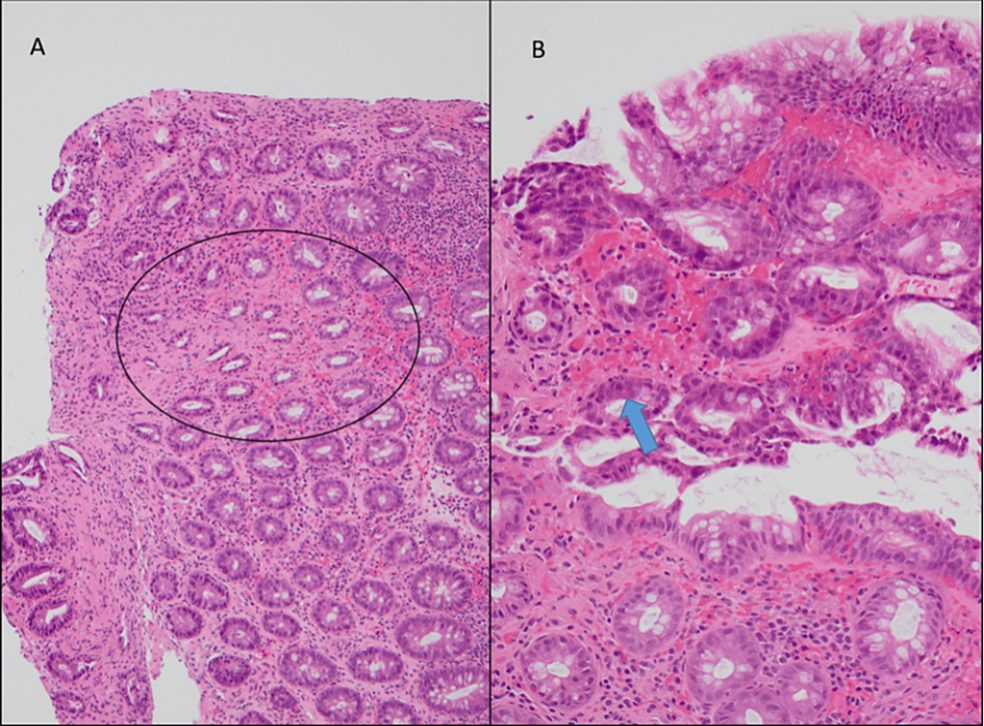
Histology slides of the sigmoid colon (A) Sigmoid colonic mucosa (H&E-stained section, original magnification ×100) showing withered crypts (circled) in contrast to the more normal crypts in the upper and lower aspects of the image. (B) Sigmoid colonic mucosa (H&E, original magnification ×200) showing acute hemorrhage in the lamina propria and focal acute inflammation with neutrophil polymorphs in the crypt epithelium (arrow).

During the procedure, biopsy samples were obtained from the lower GI tract and sent off for pathological analysis. The histological appearances were reviewed post-discharge, and they were consistent with ischemic colitis (Figure 2). In the context of clinical presentation, this was felt to be exercise induced.

Treatment and follow-up

Initial treatment focused on supportive measures, which included 3 liters of intravenous fluid for a presumed diagnosis of infective gastroenteritis. The patient was monitored as an inpatient for 48 hours, during which repeat blood tests were performed.

The patient was successfully discharged after this time, once bleeding had ceased and the patient had reestablished oral intake. Inflammatory markers had reduced and stabilized compared to the admission blood. Histology from biopsies obtained was reviewed post-discharge.

Virtual consultant follow-up occurred one week later; at this stage, symptoms had resolved other than residual fatigue. The patient was able to return to work a few days later, and over the next two months, he gradually returned to physical training. After three months, the patient returned to their normal fitness level and consistently used a cardiac monitor to ensure appropriate breaks during periods of intense physical activity.

The final follow-up took place approximately three months after admission, at which point the patient was discharged and reported no recurrence of symptoms.

## Discussion

The transient ischemic colitis caused by EIIC usually leads to a full and rapid recovery. Patient groups for whom EIIC may have special significance are those with preexisting vascular risk factors such as hypertension, peripheral vascular disease, and smoking history. It may also have increased relevance to patients with inflammatory bowel disease (IBD), where the transient pro-inflammatory effect of intense exercise regimes such as HIIT may cause a relapse in symptoms [14]. For these groups, adequate rest periods and proper hydration are strongly recommended, as is limiting intense exercise to periods of remission in IBD [15].

Beyond the abovementioned vascular risk factors, other common risk factors for EIIC include oral contraceptives [1,8] and dehydration [4,16,17], the latter being present in this case. Also relevant to this case is that coffee consumption and breakfast cereal may have worsened symptoms experienced by the patient due to their laxative effects from increased colonic motility [18] and stool water content, respectively. It is feasible that drinking coffee immediately before intense exercise may directly contribute to colonic ischemia by increasing oxygen demand (through increased colonic motility) during a period of relative hypoperfusion. This has not yet been investigated specifically and may be a topic for future research studies as a potential risk factor for EIIC.

## Conclusions

Following the COVID-19 pandemic, home exercise has become an increasingly important alternative to training in public spaces. The case presents EIIC following home-based HIIT, which is novel for both the setting of exercise and the modality of exercise causing colitis. It increases awareness of EIIC and an appreciation that any intensive aerobic exercise may lead to symptoms of transient colonic and mesenteric ischemia, particularly if suitable breaks or hydration are not incorporated into the exercise regime. We recommend actively enquiring about a history of exercise, in addition to dietary intake and travel history, when considering possible diagnoses for a presenting complaint of unexplained diarrhea.
